# Prognostic value of pathological lymph node status and primary tumour regression grading following neoadjuvant chemotherapy – results from the MRC OE02 oesophageal cancer trial

**DOI:** 10.1111/his.13491

**Published:** 2018-03-25

**Authors:** Nasser Davarzani, Gordon G A Hutchins, Nicholas P West, Lindsay C Hewitt, Matthew Nankivell, David Cunningham, William H Allum, Elizabeth Smyth, Nicola Valeri, Ruth E Langley, Heike I Grabsch

**Affiliations:** ^1^ GROW School for Oncology and Developmental Biology Department of Pathology Maastricht University Medical Centre Maastricht the Netherlands; ^2^ Department of Data Science and Knowledge Engineering Maastricht University Maastricht the Netherlands; ^3^ Section of Pathology and Tumour Biology Leeds Institute of Cancer and Pathology University of Leeds Leeds UK; ^4^ MRC Clinical Trials Unit University College London London UK; ^5^ Gastrointestinal and Lymphoma Unit Royal Marsden Hospital London UK; ^6^ Department of Surgery Royal Marsden Hospital London UK; ^7^ Department of Molecular Pathology The Institute of Cancer Research London UK

**Keywords:** oesophageal carcinoma, neoadjuvant chemotherapy, tumour regression grade

## Abstract

**Aims:**

Neoadjuvant chemotherapy (NAC) remains an important therapeutic option for advanced oesophageal cancer (OC). Pathological tumour regression grade (TRG) may offer additional information by directing adjuvant treatment and/or follow‐up but its clinical value remains unclear. We analysed the prognostic value of TRG and associated pathological factors in OC patients enrolled in the Medical Research Council (MRC) OE02 trial.

**Methods and results:**

Histopathology was reviewed in 497 resections from OE02 trial participants randomised to surgery (S group; *n* = 244) or NAC followed by surgery [chemotherapy plus surgery (CS) group; *n* = 253]. The association between TRG groups [responders (TRG1–3) versus non‐responders (TRG4–5)], pathological lymph node (LN) status and overall survival (OS) was analysed. One hundred and ninety‐five of 253 (77%) CS patients were classified as ‘non‐responders’, with a significantly higher mortality risk compared to responders [hazard ratio (HR) = 1.53, 95% confidence interval (CI) = 1.05–2.24, *P* = 0.026]. OS was significantly better in patients without LN metastases irrespective of TRG [non‐responders HR = 1.87, 95% CI = 1.33–2.63, *P* < 0.001 versus responders HR = 2.21, 95% CI = 1.11–4.10, *P* = 0.024]. In multivariate analyses, LN status was the only independent factor predictive of OS in CS patients (HR = 1.93, 95% CI = 1.42–2.62, *P* < 0.001). Exploratory subgroup analyses excluding radiotherapy‐exposed patients (*n* = 48) showed similar prognostic outcomes.

**Conclusion:**

Lymph node status post‐NAC is the most important prognostic factor in patients with resectable oesophageal cancer, irrespective of TRG. Potential clinical implications, e.g. adjuvant treatment or intensified follow‐up, reinforce the importance of LN dissection for staging and prognostication.

## Introduction

Multimodal therapy is the standard of care for many gastrointestinal malignancies.^1^ For patients with oesophageal carcinoma (OC), surgery preceded by neoadjuvant chemotherapy (NAC) or chemoradiotherapy (NACR) has proven clinical benefit, as reported in the OE02 [Medical Research Council (MRC) oesophageal working group] and CROSS (chemoradiotherapy plus surgery versus surgery alone for oesophageal or junctional cancer) trials, respectively.^2^, ^3^ While geographical variation persists in the modality of choice^4^, ^5^ and modest differential modality benefits are reported,^6^ NAC/NACR results in tumour down‐staging, increased rate of complete surgical resection and delayed recurrent and metastatic disease.^7^ To date, no randomised controlled trial (RCT) has directly compared neoadjuvant chemotherapy and neoadjuvant chemoradiation. Until the results of the Neo‐AEGIS (NEOadjuvant Trial in Adenocarcinoma of the oEsophagus and oesophagoGastric Junction International Study)^4^ trial are published, NAC remains the principal modality choice in the United Kingdom. Irrespective of neoadjuvant treatment type, the prognosis of OC patients remains poor, with a 5‐year survival of approximately 15%.^8^, ^9^ Consequently, it has been suggested that adjuvant chemotherapy or targeted therapies may be beneficial to OC patients with high‐risk disease.^8^, ^10^

As described by Mandard *et al*.^11^ and others,^12^ high‐risk patients may be identified by pathological assessment of primary tumour response [tumour regression grade (TRG)]. A prognostic value of TRG has been reported in some but not all OC studies.^13^, ^14^ Additionally, some authors report that primary TRG is not prognostic in isolation, but only when combined with lymph node (LN) status.^15^, ^16^, ^17^

Problematically, most previously reported OC studies relating to TRG suffer from methodological issues, including small patient numbers, combined data sets utilising different disease stages, use of different treatment regimens, use of different TRG systems and application of different cut‐offs to classify ‘responders’ versus ‘non‐responders’. Most importantly, previous studies have not utilised a control arm (e.g. a population of patients with the same basic characteristics treated by surgery alone) to estimate potential confounding. Thus, it is currently unclear whether pathological primary tumour TRG and/or other disease characteristics assessed in the post‐NAC resection specimen can successfully identify high‐risk OC patients,^18^ the cohort of patients which may benefit from close follow‐up or further adjuvant treatment.

Our own study assessing TRG in patients enrolled into the Medical Research Council (MRC) Adjuvant Gastric Infusional Chemotherapy (MAGIC) trial demonstrated that LN status is the only independent predictor of survival after NAC in resectable gastro‐oesophageal cancer.^7^ The majority of these patients, however, had gastric cancers; only 38 had cancers located at the gastro‐oesophageal junction or lower oesophagus. Thus, subgroup analyses relating to OC in the MAGIC trial were methodologically unfeasible.

Given such challenges, we investigated the prognostic value of Mandard TRG, LN status and other clinicopathological variables in OC patients treated by either cisplatin combined with 5‐fluorouracil (5‐FU) followed by surgery [experimental arm, chemotherapy plus surgery (CS) patients] or surgery alone (control arm, S patients) in the Phase III UK MRC OE02 randomised controlled trial of oesophageal cancer.^2^

## Materials and methods

### Ethics

The study was approved by the South East Research Ethics Committee, London, UK, REC reference: 07/H1102/111.

### Patients

A total of 802 patients with histologically or cytologically confirmed, locally advanced resectable cancer of the oesophagus were included in the MRC OE02 trial.^19^ Patients were randomised to treatment by surgery alone (S patients) or surgery preceded by pre‐operative combination chemotherapy consisting of two cycles of 5‐FU and cisplatin (CS patients).^19^ In total, 360 CS patients and 394 S patients proceeded to surgical resection; 32 CS patients and 16 S patients did not proceed to surgery for a variety of reported reasons.^19^ A small subset of patients from a single centre was exposed to preoperative radiotherapy (CS patients *n* = 26, S patients *n* = 22), according to local practice.

**Figure 1:**
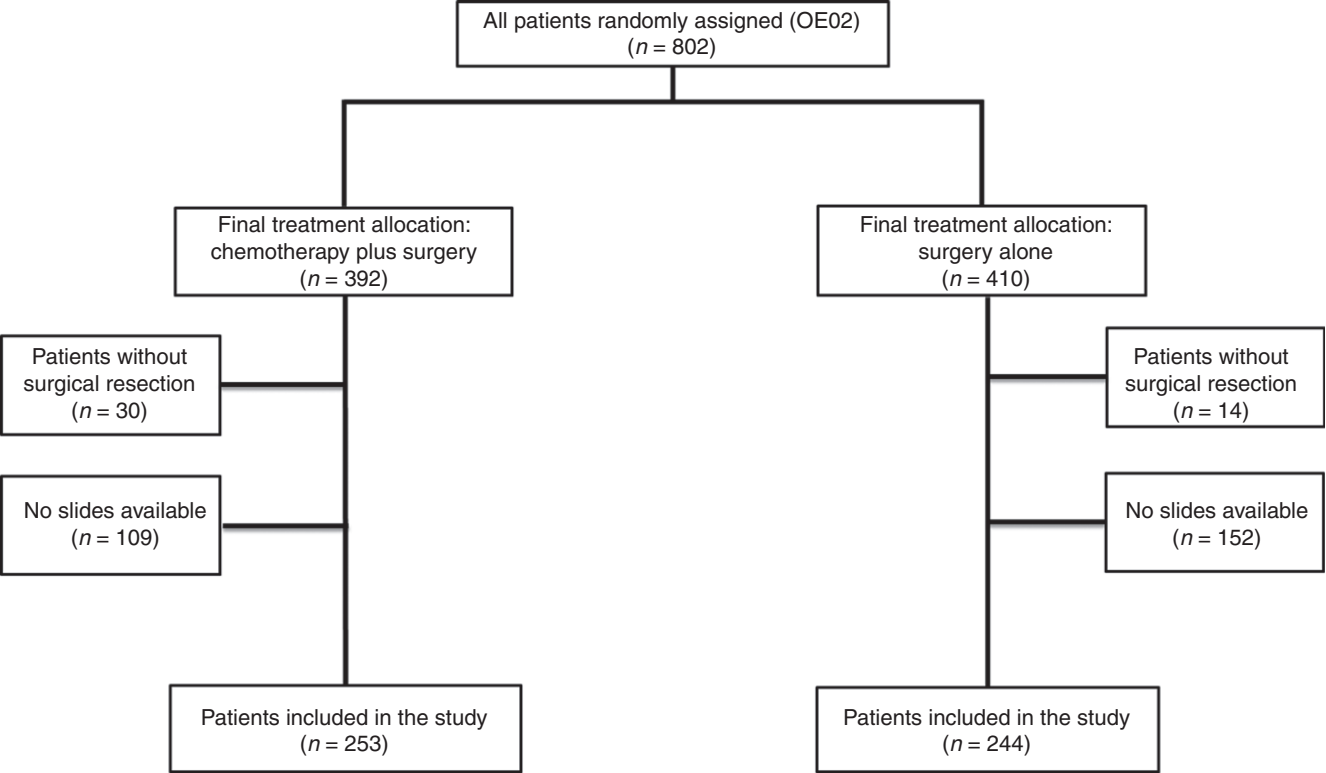
Study profile, Consolidated Standards of Reporting Trials (CONSORT).

Original haematoxylin and eosin (H&E)‐stained sections and/or blocks of the formalin‐fixed paraffin‐embedded resected specimens were collected retrospectively from 497 patients (253 CS patients and 244 S patients), equivalent to 66% of all OE02 trial patients who proceeded to surgery (Figure 1).

### Study design

All H&E‐stained sections were scanned at ×40 magnification using an Aperio XT Scanner (Aperio Technologies, Vista, CA, USA) and reviewed centrally using Aperio ImageScope digital slide viewing software (version 11.2.0.780). As no central pathology review was performed at the time of trial accrual, and TRG assessment was not included in the original pathology reporting form, histopathological assessment of the resected specimens (including primary TRG as described by Mandard *et al*.^11^) was performed centrally, with assessors blinded to treatment arm allocation.

Primary tumours from S and CS patients were assigned to the TRG/TRG‐like categories as follows: TRG1 (no evidence of residual tumour), TRG2 (fibrosis with occasional tumour cells), TRG3 (fibrosis and tumour cells with a predominance of fibrosis), TRG4 (fibrosis and tumour cells with a predominance of tumour cells) and TRG5 (tumour with no evidence of regression). For representative images of histological features for different TRG categories see Figure S1.

**Figure 2:**
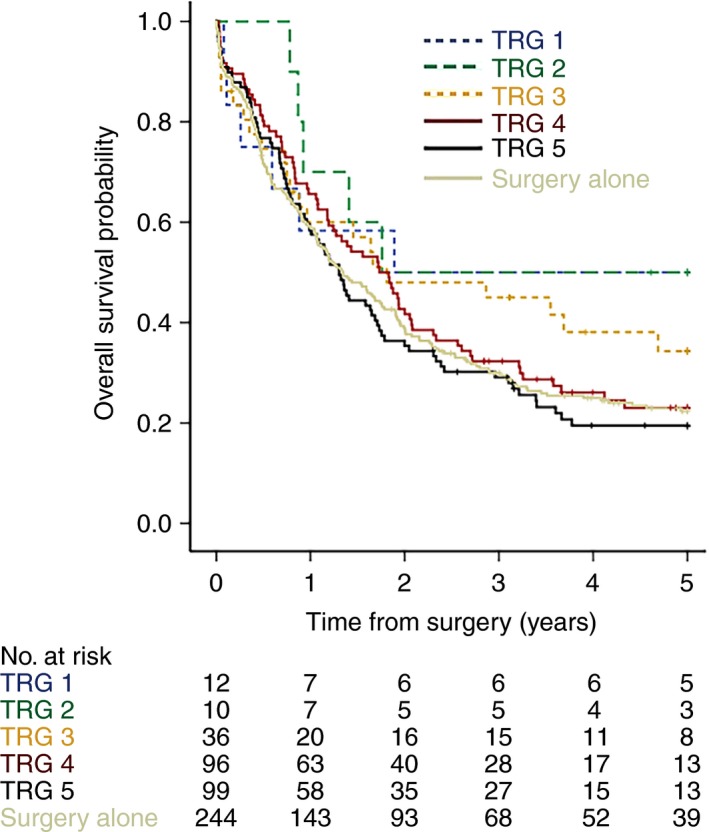
Overall survival stratified by tumour regression grade (TRG) in patients treated by neoadjuvant therapy or surgery alone. Survival of patients with TRG1, 2 or 3 tumours was similar, and markedly different from patients with TRG4 or 5 tumours. Survival of patients treated with surgery alone was similar to that of patients treated with chemotherapy plus surgery with TRG4 or 5 tumours.

Overall survival (OS) analyses were performed in CS patients using TRG and LN status (ypN) as individual variables. As initial analyses demonstrated that patients classified as TRG4 or TRG5 had at a significantly higher mortality risk when compared to those assigned to TRG1, TRG2 or TRG3 (see Results; Figure 2), patients with TRG1, TRG2 or TRG3 (TRG1–3) were classified as ‘responders’ and compared to patients with TRG4 or TRG5 (TRG4–5, ‘non‐responders’) in subsequent analyses. Furthermore, OS of patients with TRG4–5 without LN metastasis (non‐responders, LN‐negative) was compared to OS of patients with TRG4–5 and LN metastasis (non‐responders, LN‐positive). Similarly, OS of patients with TRG1–3 without LN metastasis (responders, LN‐negative) was compared to patients with TRG1–3 and LN metastasis (responders, LN‐positive).

### Clinicopathological data

The central pathology review defined histological tumour type, grade of differentiation, TRG,^11^ depth of invasion (T category) and LN status (N category), according to the International Union Against Cancer (UICC) tumour–node–metastasis (TNM) classification, 6th edition.^20^ Histopathological data that were not assessable on review of the slides, such as tumour size, tumour location, number of LNs or resection margin status, were extracted from the original pathology report. Clinical outcome data were extracted from the MRC OE02 clinical trial database.

### Statistical methods

Baseline characteristics of CS and S patients were compared using χ^2^ and Fisher's exact tests. OS time was calculated from the date of surgery to the date of death within the 5‐year follow‐up period. The surgery dates were not available for two CS patients, and the survival time was calculated from a date 9 weeks after randomisation.

Overall survival was compared using the Kaplan–Meier method and log‐rank test. Patients were stratified by individual TRG category, TRG groupings (responders versus non‐responders) and combining TRG groups with LN status (see above). In order to investigate the relationship between TRG, LN status and OS, a multivariate survival analysis was performed using a Cox proportional hazards model. A *P*‐value of <0.05 was considered significant.

## Results

Slides from 497 resection specimens (66% of OE02 trial patients undergoing surgery) were available for central pathology review (Figure 1). There were significant differences in tumour depth of invasion, LN status and tumour regression grade/tumour regression‐like changes between CS patients and S patients (Table 1, *P* = 0.003, *P* = 0.003 and *P* < 0.001, respectively).

**Table 1:** Patient characteristics

Variable	*n* (%)		*P*‐value
	Surgery (S)	Chemotherapy plus surgery (CS)	
Age category, years			
≤65	136 (55.7)	150 (59.3)	0.42
>65	108 (44.3)	103 (40.7)	
Gender			
Female	65 (26.6)	56 (22.1)	0.24
Male	179 (73.4)	197 (77.9)	
Depth of invasion [(y)pT]			
T0/Tis	0 (0)	12 (4.7)	0.003
T1	24 (9.8)	25 (9.9)	
T2	24 (9.8)	32 (12.7)	
T3	190 (77.9)	180 (71.1)	
T4	6 (2.5)	4 (1.6)	
Lymph node status [(y)pN]			
N0	81 (33.2)	117 (46.2)	0.003
N1	163 (66.8)	136 (53.8)	
(y)pTNM stage			
0	0 (0)	12 (4.8)	0.001
I	19 (7.8)	19 (7.5)	
II	77 (31.5)	97 (38.3)	
III	148 (60.7)	125 (49.4)	
Histological tumour type			
Squamous cell carcinoma	62 (28.4)	63 (26.1)	0.801
Adenocarcinoma	172 (70.5)	171 (71)	
Others	10 (4.1)	7 (2.9)	
Tumour regression grade (TRG)	‘TRG‐like changes’		
1	0 (0)	12 (4.8)	<0.001
2	3 (1.2)	10 (4)	
3	15 (6.2)	36 (14.2)	
4	74 (30.3)	96 (37.9)	
5	152 (62.3)	99 (39.1)	
TRG and lymph node status (y)pN			
TRG1–3 and N0	13 (5.3)	37 (14.6)	<0.001
TRG1–3 and N1	5 (2)	21 (8.3)	
TRG4–5 and N0	68 (27.9)	80 (31.6)	
TRG4–5 and N1	158 (64.8)	115 (45.5)	
Resection margin status			
Positive	68 (30.6)	70 (30.2)	0.91
Negative	154 (69.4)	162 (69.8)	
Tumour location			
Lower	153 (62.7)	167 (66)	0.72
Middle	63 (25.8)	61 (24.1)	
Upper	28 (11.5)	25 (9.9)	

(y)pN, lymph node metastasis; TNM, tumour–node–metastasis.

The frequency of the TRG categories in CS patients (*n* = 253) was as follows: TRG1: *n* = 12 (4.8%), TRG2: *n* = 10 (4%), TRG3: *n* = 36 (14.2%), TRG4: *n* = 96 (37.9%) and TRG5: *n* = 99 (39.1%) (Table 1). The frequency of ‘tumour regression‐like’ changes in S patients (*n* = 244) was distributed as follows: TRG1: *n* = 0 (0%), TRG2: *n* = 3 (1.2%), TRG3: *n* = 15 (6.2%), TRG4: *n* = 74 (30.3%) and TRG5: *n* = 152 (62.3%) (Table 1).

As expected, there were more patients with TRG1–3 in the CS patient group compared to the S patient group [CS, *n* = 58 (76.3%) versus S, *n* = 18 (23.7%)] and fewer patients with TRG4–5 in the CS patient group compared to the S patient group [CS, *n* = 195 (46.3%) versus S, *n* = 226 (53.7%)].

Lymph node data were available for 492 patients (CS, *n* = 250; S, *n* = 242). The median (range) number of retrieved lymph nodes was similar in both treatment arms [CS, *n* = 9 (0–67); S, *n* = 10 (0–80), *P* = 0.35].

The survival of S patients was similar to CS patients with TRG4–5 tumours [hazard ratio (HR) = 1.01; 95% confidence interval (CI) = 0.81–1.25; *P* = 0.9, Figure 2]. CS patients with TRG3 tumours demonstrated a trend towards higher mortality risk when compared to CS patients with TRG1‐2 tumours; however, this did not achieve statistical significance (HR = 1.43, 95% CI = 0.694–2.958, *P* = 0.33). Survival analyses of CS patients with TRG4–5 tumours similarly demonstrated a trend towards higher mortality risk when compared to CS group patients with TRG3 tumours, but with a slightly lower HR and also not reaching statistically significant levels (HR = 1.34, 95% CI = 0.858–2.105, *P* = 0.196).

**Figure 3:**
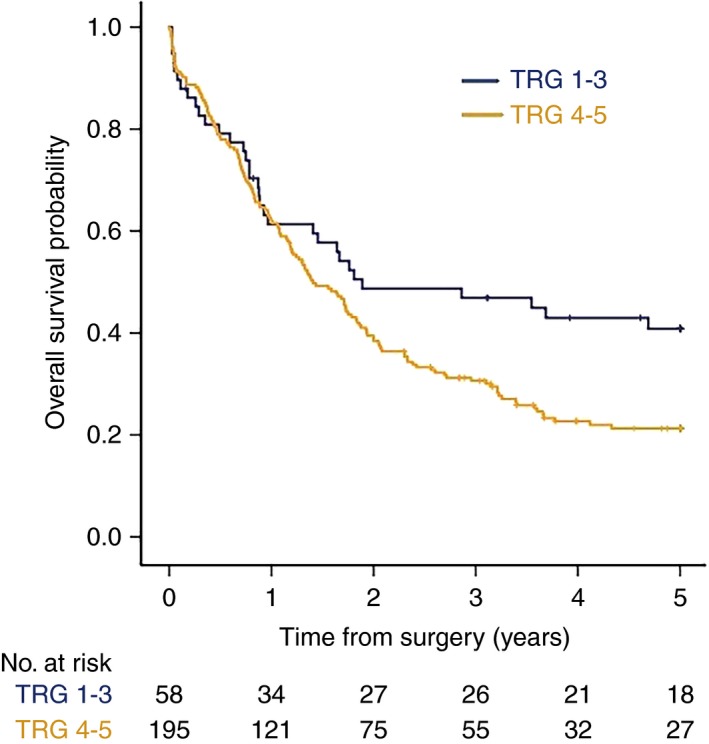
Five‐year overall survival stratified by combined tumour regression grade (TRG) in patients treated with neoadjuvant chemotherapy plus surgery in the OE02 trial. Patients with TRG1–3 tumours (responders) survived significantly longer than patients with TRG4–5 tumours (non‐responders) [hazard ratio (HR) = 1.53, 95% confidence interval (CI) = 1.05–2.24, *P* = 0.026].

The group of CS patients with TRG1–3 tumours (‘responders’) had a significantly longer OS than the group with TRG4–5 tumours (‘non‐responders) (HR = 1.53, 95% CI = 1.05–2.24, *P* = 0.026, Figures 2 and 3).

**Figure 4:**
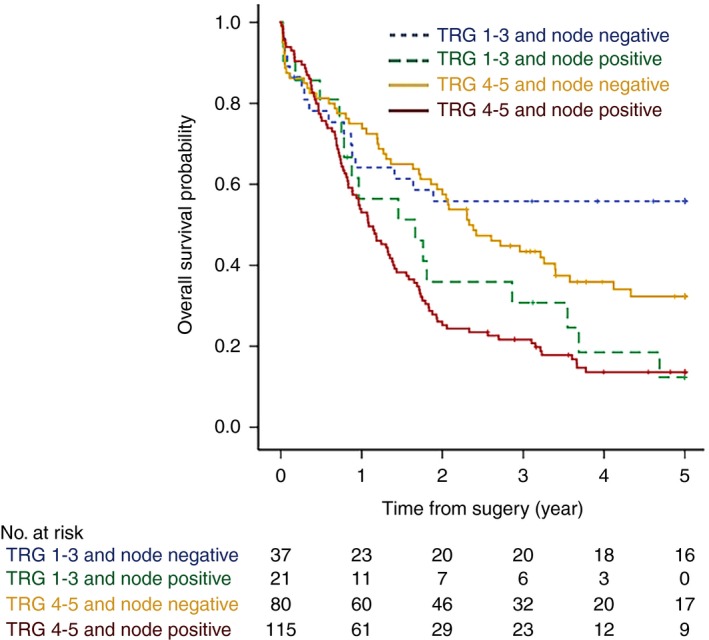
Five‐year overall survival stratified by combined tumour regression grade (TRG) and lymph node status in patients treated with neoadjuvant chemotherapy plus surgery. Responders (TRG1–3 tumours) without lymph node metastasis (node negative) survived significantly longer than responders with lymph node metastasis (node positive) [hazard ratio (HR) = 2.21, 95% confidence interval (CI) = 1.11–4.10, *P* = 0.024]. Non‐responders (TRG4–5 tumours) without lymph node metastasis (node negative) survived significantly longer than non‐responders with lymph node metastasis (node positive) (HR = 1.87, 95% CI = 1.33–2.63, *P* < 0.001). [Colour figure can be viewed at http://wileyonlinelibrary.com]

Following the combination of TRG with LN status, responders without LN metastasis survived significantly longer than responders with LN metastasis (HR = 2.21, 95% CI = 1.11–4.10, *P* = 0.024). Similarly, non‐responders without LN metastasis survived significantly longer than non‐responders with LN metastasis (HR = 1.87, 95% CI = 1.33–2.63, *P* < 0.001, Figure 4). Furthermore, the survival of responders without LN metastasis was similar to that of non‐responders without LN metastasis (*P* = 0.12), and the survival of responders with LN metastasis was similar to that of non‐responders with LN metastasis (*P* = 0.49).

Multivariate analyses which incorporated TRG and LN status into the model demonstrated that LN status was the only independent factor predictive of survival in CS patients (ypN1; HR = 1.93, 95% CI = 1.42–2.62, *P* < 0.001), with assignment to TRG4–5 (‘non‐responders’) not influencing survival significantly (HR = 1.35, 95% CI = 0.92–1.98, *P* = 0.12, Table 2).

**Table 2:** Multivariate analysis of factors affecting 5‐year overall survival in patients treated with neoadjuvant chemotherapy plus surgery (*n* = 253)

Variable	All patients (*n* = 253)	
	HR (95% CI)	*P*‐value
TRG1–3		
TRG4–5	1.35 (0.92, 1.98)	0.12
ypN0		
ypN1	1.93 (1.42, 2.62)	<0.001

TRG, tumour regression grade; ypN, lymph node status after chemotherapy; HR, hazard ratio; CI, confidence interval.

Following exclusion of patients exposed to pre‐operative radiotherapy (CS patients *n* = 26, S patients *n* = 22), exploratory subgroup analyses revealed similar prognostic associations to those from the analysis of the whole cohort in terms of magnitude and direction of effect (Figures S2–S5).

## Discussion

Following publication in 2002, results from the MRC OE02 trial changed clinical practice for patients with locally advanced resectable OC by establishing neoadjuvant combination chemotherapy followed by surgery as standard of care.^19^ Tumour regression grading was, however, not reported in the original OE02 trial,^19^ nor in the report of long‐term follow‐up for OE02,^2^ nor in any other large multicentre randomised trial using 5‐FU/cisplatin‐based neoadjuvant chemotherapy in OC patients. Thus, to date, the potential clinical value of TRG in OC patients remains unclear.^7^, ^21^, ^22^ Recently, our group has demonstrated, utilising material from patients with resectable gastric cancer enrolled into the MRC MAGIC trial, that postoperative LN status, not TRG, is an independent predictor of survival following NAC.^7^ In an effort to define the clinical value of TRG in a pure OC cohort, we centrally reviewed (and assigned TRG categories to) material from 497 patients with resectable OC enrolled in OE02, a large randomised control trial that allocated patients either to NAC followed by surgery or surgery alone.^2^, ^19^

Overall, our present study shows similar results to the MAGIC trial of gastric cancer patients.^7^ The very low number of patients treated by surgery alone showing tumour regression‐like changes is concordant with MAGIC trial data and supports the accuracy of subjective TRG. It is noteworthy that the Kaplan–Meier overall survival plots for similar TRG group assignments tracked differently when comparing MAGIC^7^ and OE02 trial patients (Figure 2). The reasons for this are unknown but, for methodological consistency, we utilised a similar approach as used in the MAGIC trial of merging groups based on similar survival profiles. Thus, patients classified as TRG1 and TRG2 were grouped as ‘responders’ in MAGIC^7^ whereas, in the present study, TRG1, 2 and 3 cohorts were grouped as ‘responders’.

In the present study, patients with lymph node metastases following NAC treatment had the worst overall survival, a feature associated with a doubling of mortality risk when compared to patients without lymph node metastases. Thus, although the prognostic implication of TRG measurement was demonstrable in univariate analysis, the adverse prognostic effect of lymph node metastases was retained irrespective of the perceived response of the primary tumour to treatment. Our study emphasises the importance of a complete and thorough lymph node dissection by the surgeon, as well as comprehensive retrieval of lymph nodes from the resected specimen by the pathologist. While TRG grading may offer some information on the local response to treatment, the lymph node status after NAC appears to be more important in identifying high‐risk OC patients and thus may potentially direct clinical management decisions in OC patients with high‐risk disease for whom adjuvant therapy and/or intensified follow‐up is probably warranted.

Our study has some limitations. This is a retrospective study based on a 66% subset of OE02 trial patients who underwent surgery. As the trial was conducted between 1992 and 1998, and material collection initiated 20 years following closure of the trial, material was not obtainable for all patients for central review. We were, however, able to obtain TRG values from almost double the number of patients in comparison to the MAGIC trial study,^7^ increasing the statistical power for the comparison of the prognostic importance of TRG and LN status in subgroup analyses.

The number of lymph nodes could not always be verified during the central pathology review due to lack of documentation within the pathology reports, but is lower than expected in more contemporary trials reflecting clinical practice at the time of the trial. For the majority of patients, the number of positive lymph nodes was not independently verifiable during central review, as information regarding what was included in each paraffin block (the blocking list) was often lacking. Additionally, anatomical location of the nodes was not recorded in most cases. This latter aspect is probably important, as recently published studies suggest that treatment response in lymph nodes and anatomical location of lymph nodes with residual disease may be more relevant than primary tumour TRG when predicting patient prognosis.^23^, ^24^, ^25^ Primary analyses also included a small proportion of patients (CS *n* = 26, S *n* = 22), derived from a single centre, who were subjected to pre‐operative radiotherapy according to local practice. We performed exploratory subgroup analyses excluding these patients, demonstrating the retention of the prognostic effects observed in the entire cohort, some with enhanced prognostic effects (Figures S2–S5). This subgroup also demonstrated borderline statistical significance for TRG as an independent prognostic factor; however, such an interpretation should be treated with caution, as these demonstrated good TRG response but poor survival, thus potentially skewing statistical interpretation.

Tumour regression grading systems categorise regressive changes following chemotherapy in order to provide prognostic information on the basis of characteristic histopathological changes. As reviewed recently by Langer and Becker, a variety of systems are reported for application in gastrointestinal malignancies, each with relative advantages and disadvantages.^26^ Despite reported disadvantages such as poor reproducibility, the Mandard system, as utilised for this study, has demonstable equivalence of reproducibility when compared to other systems, including the four‐tier Becker system, specifically within the context of oeosphageal malignancies.^27^

In summary, this study, to the best of our knowledge, represents the first report of the potential prognostic value of centrally reviewed TRG, lymph node status and other histopathological variables in a randomised trial population of patients with locally advanced oesophageal cancer treated either by neoadjuvant combination chemotherapy followed by surgery or surgery alone. We have demonstrated that pathological LN status is the principal determinant of OC patient survival after neoadjuvant chemotherapy. The clinical implications of potential adjuvant treatment or intensified surveillance reinforce the importance of meticulous lymph node dissection by the surgeon and the pathologist. Further work is needed to demonstrate if TRG of intranodal deposits and the anatomical location of lymph nodes with and without regression (implying heterogeneity of nodal response) can provide clinically relevant information beyond the number of positive lymph nodes alone.

## Conflicts of interest

D.C. acknowledges funding support of AstraZeneca, Amgen, Bayer, Celgene, Merrimack, Merck Serono, MedImmune, Sanofi. W.A. acknowledges funding from Eli Lilly and Nestle. R.L. acknowledges funding from Bayer. N.W. acknowledges funding from Eisai. N.D., G.H., L.H., M.N., E.S., N.V. and H.G. have no associations to declare.

## References

[1] Thies S , Langer R . Tumor regression grading of gastrointestinal carcinomas after neoadjuvant treatment. Front. Oncol. 2013; 3; 262. 24109590 10.3389/fonc.2013.00262 PMC3791673

[2] Allum WH , Stenning SP , Bancewicz J , Clark PI , Langley RE . Long‐term results of a randomized trial of surgery with or without preoperative chemotherapy in esophageal cancer. J. Clin. Oncol. 2009; 27; 5062–5067. 19770374 10.1200/JCO.2009.22.2083

[3] van Hagen P , Hulshof MC , van Lanschot JJ *et al* Preoperative chemoradiotherapy for esophageal or junctional cancer. N. Engl. J. Med. 2012; 366; 2074–2084. 22646630 10.1056/NEJMoa1112088

[4] Reynolds J , Preston S , O'Neill B *et al* Icorg 10‐14: Neoadjuvant Trial in Adenocarcinoma of the Oesophagus and oesophagogastric Junction International Study (NEO‐AEGIS). BMC Cancer 2017; 17; 401. 28578652 10.1186/s12885-017-3386-2 PMC5457631

[5] Lordick F , Mariette C , Haustermans K , Obermannova R , Arnold D ; ESMO Guidelines Committee . Oesophageal cancer: ESMO clinical practice guidelines for diagnosis, treatment and follow‐up. Ann. Oncol. 2016; 27; v50–v57. 27664261 10.1093/annonc/mdw329

[6] Markar SR , Noordman BJ , Mackenzie H *et al* Multimodality treatment for esophageal adenocarcinoma: multi‐center propensity‐score matched study. Ann. Oncol. 2017; 28; 519–527. 28039180 10.1093/annonc/mdw560 PMC5391716

[7] Smyth EC , Fassan M , Cunningham D *et al* Effect of pathologic tumor response and nodal status on survival in the Medical Research Council adjuvant gastric infusional chemotherapy trial. J. Clin. Oncol. 2016; 34; 2721–2727. 27298411 10.1200/JCO.2015.65.7692 PMC5019747

[8] Pennathur A , Gibson MK , Jobe BA , Luketich JD . Oesophageal carcinoma. Lancet 2013; 381; 400–412. 23374478 10.1016/S0140-6736(12)60643-6

[9] Cancer Research UK . Oesophageal cancer survival statistics. London, UK: Cancer Research UK, 2017.

[10] Brescia AA , Broderick SR , Crabtree TD *et al* Adjuvant therapy for positive nodes after induction therapy and resection of esophageal cancer. Ann. Thorac. Surg. 2016; 101; 200–208; discussion 208–210. 26507424 10.1016/j.athoracsur.2015.09.001 PMC4745588

[11] Mandard AM , Dalibard F , Mandard JC *et al* Pathologic assessment of tumor regression after preoperative chemoradiotherapy of esophageal carcinoma. Clinicopathologic correlations. Cancer 1994; 73; 2680–2686. 8194005 10.1002/1097-0142(19940601)73:11<2680::aid-cncr2820731105>3.0.co;2-c

[12] Becker K , Mueller JD , Schulmacher C *et al* Histomorphology and grading of regression in gastric carcinoma treated with neoadjuvant chemotherapy. Cancer 2003; 98; 1521–1530. 14508841 10.1002/cncr.11660

[13] Guo K , Cai L , Zhang Y *et al* The predictive value of histological tumor regression grading (TRG) for therapeutic evaluation in locally advanced esophageal carcinoma treated with neoadjuvant chemotherapy. Chin. J. Cancer 2012; 31; 399–408. 22572013 10.5732/cjc.011.10406 PMC3777510

[14] Meredith KL , Weber JM , Turaga KK *et al* Pathologic response after neoadjuvant therapy is the major determinant of survival in patients with esophageal cancer. Ann. Surg. Oncol. 2010; 17; 1159–1167. 20140529 10.1245/s10434-009-0862-1

[15] Schneider PM , Baldus SE , Metzger R *et al* Histomorphologic tumor regression and lymph node metastases determine prognosis following neoadjuvant radiochemotherapy for esophageal cancer: implications for response classification. Ann. Surg. 2005; 242; 684–692. 16244542 10.1097/01.sla.0000186170.38348.7b PMC1409844

[16] Samson P , Robinson C , Bradley J *et al* Neoadjuvant chemotherapy versus chemoradiation prior to esophagectomy: impact on rate of complete pathologic response and survival in esophageal cancer patients. J. Thorac. Oncol. 2016; 11; 2227–2237. 27544058 10.1016/j.jtho.2016.07.031 PMC5118087

[17] Parry K , van Rossum PS , Haj Mohammad N , Ruurda JP , van Hillegersberg R . The effect of perioperative chemotherapy for patients with an adenocarcinoma of the gastroesophageal junction: a propensity score matched analysis. Eur. J. Surg. Oncol. 2017; 43; 226–233. 27424786 10.1016/j.ejso.2016.06.393

[18] Noble F , Nolan L , Bateman AC *et al* Refining pathological evaluation of neoadjuvant therapy for adenocarcinoma of the esophagus. World J. Gastroenterol. 2013; 19; 9282–9293. 24409055 10.3748/wjg.v19.i48.9282 PMC3882401

[19] Medical Research Council Oesophageal Cancer Working Group . Surgical resection with or without preoperative chemotherapy in oesophageal cancer: a randomised controlled trial. Lancet 2002; 359; 1727–1733. 12049861 10.1016/S0140-6736(02)08651-8

[20] Sobin LH , Wittekind C . International Union Against Cancer (UICC): TNM classification of malignant tumors. 6th ed New York: Wiley, 2002.

[21] Hatogai K , Fujii S , Kojima T *et al* Prognostic significance of tumor regression grade for patients with esophageal squamous cell carcinoma after neoadjuvant chemotherapy followed by surgery. J. Surg. Oncol. 2016; 113; 390–396. 27100024 10.1002/jso.24151

[22] Langer R , Ott K , Feith M , Lordick F , Siewert JR , Becker K . Prognostic significance of histopathological tumor regression after neoadjuvant chemotherapy in esophageal adenocarcinomas. Mod. Pathol. 2009; 22; 1555–1563. 19801967 10.1038/modpathol.2009.123

[23] Philippron A , Bollschweiler E , Kunikata A *et al* Prognostic relevance of lymph node regression after neoadjuvant chemoradiation for esophageal cancer. Semin. Thorac. Cardiovasc. Surg. 2016; 28; 549–558. 28043475 10.1053/j.semtcvs.2016.04.003

[24] Bollschweiler E , Holscher AH , Metzger R *et al* Prognostic significance of a new grading system of lymph node morphology after neoadjuvant radiochemotherapy for esophageal cancer. Ann. Thorac. Surg. 2011; 92; 2020–2027. 22115212 10.1016/j.athoracsur.2011.06.091

[25] Nieman DR , Peyre CG , Watson TJ *et al* Neoadjuvant treatment response in negative nodes is an important prognosticator after esophagectomy. Ann. Thorac. Surg. 2015; 99; 277–283. 25442991 10.1016/j.athoracsur.2014.07.037

[26] Langer R , Becker K . Tumor regression grading of gastrointestinal cancers after neoadjuvant therapy. Virchows Arch. 2018; 472; 175–186. 28918544 10.1007/s00428-017-2232-x

[27] Karamitopoulou E , Thies S , Zlobec I *et al* Assessment of tumor regression of esophageal adenocarcinomas after neoadjuvant chemotherapy: comparison of 2 commonly used scoring approaches. Am. J. Surg. Pathol. 2014; 38; 1551–1556. 25140894 10.1097/PAS.0000000000000255

